# Institutional delivery and associated factors among women who gave birth in Benishangul Gumuz region, South West Ethiopia

**DOI:** 10.3389/fpubh.2022.965524

**Published:** 2022-12-09

**Authors:** Temesgen Arega, Teshale Mulatu, Afework Alemayehu, Ibsa Mussa, Merga Dheresa

**Affiliations:** ^1^Mandura District Health Office, Beneshangul Gumuz, Asosa, Ethiopia; ^2^School of Nursing and Midwifery, College of Health and Medical Science, Haramaya University, Harar, Ethiopia; ^3^School of Public Health, College of Health and Medical Sciences, Haramaya University, Harar, Ethiopia

**Keywords:** predictors, institutional delivery, Mandura, Benishangul Gumuz, Ethiopia

## Abstract

**Background:**

Maternal mortality from pregnancy and childbirth remains a major public health problem. Increasing access to institutional delivery is one of the key strategies to reduce childbirth-related maternal mortality. Despite all the efforts, institutional deliveries in Ethiopia remain low. Understanding factors associated with institutional delivery is important to devise strategies to improve facility based child birth. Hence, this study assessed the prevalence of institutional delivery and associated factors to bridge the gap.

**Methods:**

A community-based cross-sectional study was employed from March-April 2020. Multi-stage sampling was employed to select 500 mothers who gave birth within the last 12 months in Mandura district, Benishangul Gumuz Region, Ethiopia. Data were collected using pre tested structured questionnaire through face-to-face interview. Logistic regression models were fitted to assess the predictors of institutional delivery. Adjusted Odds ratios with 95% CI was used to show associations and statistical significance was set at a *p* < 0.05.

**Results:**

This study indicated that the prevalence of institutional delivery was 28.8% CI (25–33.3%). Having a positive attitude (AOR = 9.6,95%CI:2.5–35.9), attending antenatal care (ANC) at least once (AOR = 16.1,95%CI:9.6–22), attending ANC more than three times (AOR = 17.2, 95% CI:13.5–43.8), having good knowledge (AOR = 11.1, 95%CI: 2.7–45.4), and facing complications during pregnancy (AOR = 4.04, 95%CI: 1.0–16.0) were significantly associated with institutional delivery.

**Conclusion:**

The prevalence of institutional delivery in this study was low. Positive attitude toward institutional delivery, attending ANC, having good knowledge about institutional delivery, and facing complications during pregnancy were identified predictors of institutional delivery. Strategies with a focus on increasing ANC uptake, improving mothers' knowledge, and promoting institutional delivery at the community level are critical.

## Background

Maternal mortality from pregnancy and child birth remains a major public health problem globally. Approximately 295,000 women died in 2017; related to pregnancy and childbirth. The large number of these deaths (94%) happened in developing countries, and Sub-Saharan Africa alone accounted for roughly two-thirds (196,000) of maternal deaths (1, 2).

Averting maternal death remains the priority agenda of Sustainable Development Goal (SDG-3) with the plan to reduce maternal mortality ratio (MMR) to global target of 70 per 100,000 livebirths or less by 2030. However, maternal mortality worldwide dropped by 38% between 2000 and 2017; with this progress, the SDG-3 will not be achieved at the expense of millions of lives (1, 3).

Globally more than three fourth of maternal deaths are due to direct obstetric complications which includes hemorrhage, infection, complication of unsafe abortion, pregnancy induced hypertension and obstructed labor. Post-partum hemorrhage alone accounts for 25% of maternal death with the vast majority occurring within 24 h after delivery (4, 5). Most of these deaths could have been averted if the women have received basic maternal health care from skilled care provider.

Increasing access to institutional delivery is one key approach to reduce maternal deaths related to childbirths in developing countries, such as Ethiopia. Institutional delivery warrants safe childbirth, reduce post-partum complications, enhance survival ‘of mothers and newborns. It is predicted that delivery at health facilities could decrease 16–33% of maternal death (4, 6).

Ethiopia is one of the big contributors of global maternal death among 10 countries that comprised 58% of maternal deaths following India, Nigeria, and Democratic Republic of Congo (7). The Ethiopian demographic health survey report indicated that maternal mortality rate dropped by 39% from 676 in 2011 to 412 per 100,000 live births in 2016 (8, 9). More ever, the proportion of skilled birth attendance have been increased from 28 % in 2016 to 48% in 2019 and still majority of the women are giving birth at home without any skilled professional assistance (9, 10).

Studies showed that numerous factors affect institutional delivery services. Socioeconomic/demographic factors, health facility factors, obstetric factors, maternal preference of birth places, knowledge about the benefits of institutional delivery, knowledge about pregnancy danger signs, pregnancy plan, family size, birth preparedness and complication readiness are some factors influencing delivery service utilization (11–13).

To achieve the target of SDGs, Ethiopian government has developed various interventions to improve access to maternal health services. For instance, deployment of health extension workers (HEWs) working in the rural set up across all regions of the country to provide basic maternal health services (14). In addition, federal ministry of health (FMOH) put a national goal to scale up facility-based childbirth to 70% by 2025 (15). Despite all these efforts, institutional deliveries remain low.

Although institutional delivery has been widely investigated in Ethiopia, most of the studies relied on the data from national/regional surveys whereas studies on predictors of institutional delivery in the particular study area were limited. Understanding factors associated with institutional delivery in particular set up is important to devise strategies to improve facility based child birth in the study area. Thus, this study assessed prevalence and associated factors of institutional delivery among mothers who gave birth in Mandura district, Benishangul Gumuz Region, Ethiopia.

## Methods and material

### Study design, setting and period

A community based cross-sectional study was carried out among women who gave birth in Mandura district.

Mandura is one of the districts or woredas found in the Metekel Zone of Benishangul-Gumuz Region in Ethiopia; located 546 KM from Addis Ababa. According to 2007 national survey this woreda had a total population of 40,746, of whom 21,241 were men and 19,505 were women; 45% (8776) were reproductive age groups. The district had 3 urban and 17 rural kebeles (small administrative units in the district) (16). The study was conducted from March-April 2020.

### Source and study population

All reproductive age group women residing in Mandura district were the source population where as those women who gave birth within the last 12 months in the selected kebeles of the study area were study population. Critically ill mothers who were unable to respond were excluded from the study.

### Sample size determination and sampling technique

The sample size was determined using single population formula; *n* = (Zα/2)2 × P (1 - p)/d2 with the following assumptions: The proportion of institutional delivery P = 18% (17), degree of precision (d) = 0.05, 95% confidence interval, and design effect of 2. Considering 10% non-response rate, the final sample size was 500. Multi-stage sampling technique was used to select nine kebeles (three urban kebeles and six rural kebeles) in the district. The kebeles were selected by lottery method. Proportional allocation was made to give equal chance for each kebele. All eligible households from each kebele were selected by systematic random sampling. The list of households was obtained from the health post (health extension workers). If more than one eligible woman were in the selected household, only one of them was interviewed.

### Data collection **instrument and procedures**

Data were collected using a structured questionnaire through face-to-face interview. The questionnaire was prepared by reviewing various literatures (17–24). The questionnaire contains socio-demographic characteristics, health facility/service-related factors, obstetric characteristics and participants' knowledge and attitude toward institutional delivery. Four health extension workers and two Bsc midwives were recruited for data collection and supervision. A two-day training was given for data collectors and supervisors on the contents of the tools and aim of the study. The pretest was conducted on 5% of the sample (25 participants) outside the study area. Some modifications and adjustments were made to questions based on lessons obtained during the pretest. The collected data was checked daily by supervisors and principal investigators for its completeness and consistency.

### Operational definitions

#### Institutional delivery

Refers to if the woman gave birth at were minimal facilities and skilled professionals to diagnose, manage or refer obstetric complications are available (13, 18).

#### Knowledge about institutional delivery

The woman was categorized as having good knowledge if she scored 50% and above for knowledge questions, otherwise poor knowledge (13, 18).

#### Attitude toward institutional delivery

The women was considered as having positive attitude toward institutional delivery, if she scored 50% and above on attitude questions and negative attitude if not (18).

### Data processing and analysis

Data were entered into computer, cleaned and coded using Epi-Data 3.1, then exported to SPSS version 22 for analysis. Descriptive statistics were computed to determine the frequencies and means. Model fitness was checked using Hosmer and Lemeshow's test for goodness of fit. Binary logistic regression analysis was performed to identify predictors of institutional delivery. Variables with *p* < 0.25 in bivariable analysis were retained into multivariable logistic regression model and adjusted odds ratio with 95% confidence interval was used to measure strength of association. Statistical significance was set at *p* < 0.05.

### Ethical consideration

The ethical approval for the study was sought from Institutional Health Research Review Committee of Haramaya University, College of health and medical science. Voluntary, written and signed consent was obtained from study participants, from a parent and/or legal guardian for the participants under 18, and from legally authorized representatives for illiterate participants. Confidentiality was maintained and all basic rights of participants were respected.

## Results

### Socio-demographic characteristics of study participants

A total of 500 women participated in the study, with 100% response rate. The mean age of study participants was 28.85(SD ± 5.79). Majority (93.8%) of the respondents were married and from rural 377 (75.4%). The highest proportion 366 (73.2%) of study participants were illiterate (Table 1).

**Table 1:** Socio-demographic characteristics of women who gave birth in the past 12 months in Mandura district, Benishangul Gumuz region, Ethiopia, 2020 (*n* = 500).

**Variable**	**Category**	**Frequency**	**Percent (%)**
Age of mother	15–19	22	4.4
	20–24	93	18.6
	25–29	165	33.0
	30–34	125	25.0
	35–39	69	13.8
	≥40	26	5.2
Mean age	28.85 (SD± 5.79)		
Residence	Urban	123	24.6
	Rural	377	75.4
Marital status	Married	469	93.8
	Divorced	15	3.0
	Widowed	6	1.2
	Single	6	1.2
	Separated	4	8
Religion	Orthodox	363	72.6
	Muslim	76	15.2
	Protestant	31	6.2
	Catholic	30	6.0
Ethnicity	Gumuz	362	72.4
	Agaw	73	14.6
	Amhara	46	9.2
	Oromo	10	2.0
	Shinasha	9	1.8
Maternal occupation	House wives	218	43.6
	Government employer	36	7.2
	Private employer	10	2.0
	Farmer	172	34.4
	Merchant	29	5.8
	Daily laborer	13	2.6
	Student	22	4.4
Maternal education	Illiterate	366	73.2
	Primary school	85	17.0
	Secondary school and above	49	9.8

### Obstetric characteristics of the respondents

More than one third 176 (35.2%) of study participants attended ANC for their current pregnancy and 139(27.8%) of participants reported that their current pregnancy was planned. More than half 291(58.2%) of women had poor knowledge about institutional delivery, whereas 365(73%) of them had negative attitude toward institutional delivery (Table 2).

**Table 2:** Obstetric conditions of women who gave birth in the past 12-months in Mandura district, Benishangul Gumuz region, Ethiopia, 2020 (*n* = 500).

**Variables**	**categories**	**Frequency**	**Percent (%)**
ANC visit	No	324	64.80
	Yes	176	35.2
Number of ANC visits	1–2 visit	79	15.80
	3–4 visit	97	19.40
Pregnancy status	Not planned	361	72.20
	Planned	139	27.80
Complications during current pregnancy	No	345	69.00
	Yes	155	31.00
Knowledge about institutional delivery	Good	209	41.80
	Poor	291	58.20
Knowledge about complication	Good	265	53.00
	Poor	235	47.00
Attitude toward delivery service at HF	Positive	135	27.00
	Negative	365	73.00
Distance from HF in hrs	≤ 1 h	171	34.20
	>1 h	329	65.80

ANC, Antenatal care; HF, Health facility.

### Institutional delivery among study participants

**Figure 1:**
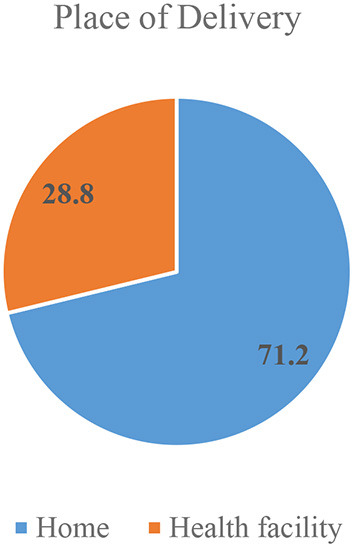
Place of delivery among women who gave birth in the past 12 months in Mandura district, Benishangul Gumuz, Ethiopia, 2020 (*n* = 500).

The prevalence of institutional delivery in this study was 28.8% (95% CI: 25, 33.2%) (Figure 1).

**Figure 2:**
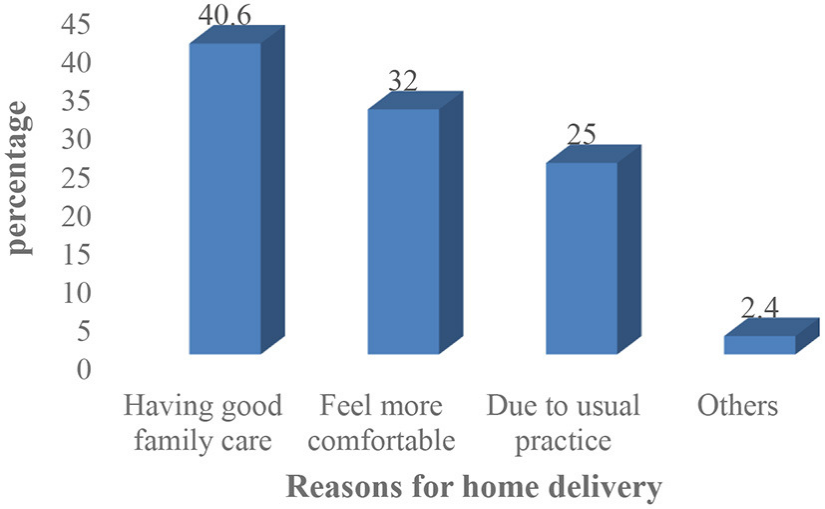
Reasons for home delivery in Mandura district, Benishangul Gumuz, Ethiopia, 2020 (*n* = 356).

With regard to place of delivery a significant number of participants 356(71.2%) delivered at home. The major reported reasons for home delivery were: feel more comfortable at home (32%), good family care at home (40.6%), previous usual practice (25%) and others (2.4%) %) (Figure 2).

### Predictors of institutional delivery

The multi-variable analysis showed that complications faced during pregnancy, knowledge about institutional delivery, attitude toward institutional delivery and antenatal care visit were strong predictors of institutional delivery. Women who faced complications during current pregnancy were 4.04 times more likely to give birth at health facility compared to their counterparts (AOR = 4.04, 95% CI:1.02–16.04, *P* = 0.04). Mothers with at least one ANC visit during the recent pregnancy were 16.1 times more likely to give birth at health facility compared to those who had no ANC follow up (AOR = 16.1, 95% CI:9.6–22, *P* = 0.001). The odds of institutional delivery were 11.1 times higher among mothers who had good knowledge compared to those who had poor knowledge (AOR = 11.1, 95% CI: 2.7–45.4, *P* = 0.001). The odds of institutional delivery were 9.6 times higher among mothers with positive attitude than mothers who had negative attitude toward child birth at HF (AOR = 9.6, 95% CI: 2.5–35.9, *P* = 0.001) (Table 3).

**Table 3:** Factors associated with institutional delivery among mothers who gave birth in the past 12 months in Mandura district, Benishangul Gumuz, Ethiopia, 2020 (*n* = 500).

**Variables**	**Categories**	**Place of delivery**				***p*-value**
		**Home (%)**	**Health facility (%)**	**COR (95%CI)**	**AOR (95%CI)**	
Residence	Urban	41 (33.3%)	82 (66.7%	10.1 (6.3–16.15)	0.90 (0.11–7.12)	0.92
	Rural	315 (83.6%)	62 (16.4%	1	1	
Maternal education	Illiterate	314 (85.8%)	52 (14.2%)	1	1	
	Primary school	38 (44.7%)	47 (55.3%)	0.15 (0.05–0.43)	6.07 (0.02–1.37)	0.32
	Secondary school and above	4 (8.2%)	45 (91.8%)	0.11 (0.36–0.33)	0.22 (0.01–2.65)	0.23
Complication faced during pregnancy	No	215 (62.3%)	130 (37.7%)	1	1	
	Yes	141 (91.0%)	14 (9.0%)	6.09 (3.3–10.9)	4.04 (1.02–16.0) *	0.04
Knowledge about institutional delivery	Good	78 (37.3%)	131 (62.7%)	35.0 (19.2–66.9)	11.1 (2.7–45.4) *	0.001
	Poor	278 (95.5%)	13 (4.5%)	1	1	
Knowledge about complication	Good	134 (50.6%)	131 (49.4%)	16.6 (9.0–30.6)	2.14 (0.48–9.56)	0.31
	Poor	222 (94.5%)	13 (5.5%)	1		
Attitude toward delivery service at HF	Positive	15 (11.1%)	120 (88.9%)	113.6 (57–223)	9.6 (2.5–35.9) *	0.001
	Negative	341 (93.4%)	24 (6.6%)	1	1	
last pregnancy planned	No	281 (77.8%)	80 (22.2%)	1	1	
	Yes	75 (54.0%)	64 (46.0%)	2.9 (1.9–4.5)	1.72 (0.50–5.89)	0.38
Number of ANC visit	No visit	319 (98.5%)	5 (1.5%)	1	1	
	1–2 visit	21 (26.6%)	58 (73.4%)	17.6 (6.3–48.6)	16.1 (9.6–22) *	0.001
	3–4 visit	16 (16.5%)	81 (83.5%)	32.2 (11.4–90.7)	17.2 (13.5–43.8) *	0.001
Distance of HF from home (in hr)	≤ 1 h	139 (81.3%)	32 (18.7%)	2.2 (1.4–3.5)	0.51 (0.11–2.37)	0.39
	>1 h	217 (66.0%)	112 (34.0%)	1	1	

^*^P < 0.05.

## Discussion

This study assessed institutional delivery and associated factors among women who gave birth in Mandura District of Benishangul gumuz, Ethiopia. The study revealed the prevalence of institutional delivery was 28.8% (95% CI: 25–33.3%). Good knowledge about institutional delivery, positive attitude toward institutional delivery, complication faced during pregnancy, and having ANC follow up were found to be significant predictors of institutional delivery.

The prevalence of institutional delivery in this study is consistent with that of a study done in Cheha district, Gurage zone, SNNPR (31%), systematic review report from Ethiopia (31%) and other national study in Ethiopia (26.2%) (19–21). But it is higher than studies conducted in Liban District (13.9%), Guji Zone, Oromia, Southern Ethiopia (22), Dangla (18.3%), Northern Ethiopia (23), and Awi zone (18.8%), North West Ethiopia (24). The difference might be due to the length of time interval between the studies. At present there is improved exposure to health education and increased access to maternal health care services with the involvement of health extension workers.

However, this study finding was lower compared to Ethiopian Demographic Health Survey report (EDHS, 2019) which was 48% (10), studies conducted at Arbaminch Town, southern Ethiopia 73.2% (25), Woldia Town, Ethiopia 74.7% (26) and Bench maji Zone, South West Ethiopia 78.30% (27). The difference might be explained by sample size difference, sociodemographic variation, study setting and accessibility of the service.

This study revealed that women who faced complications during current pregnancy were 4 times (AOR = 4.04, 95% CI:1.02–16.04) more likely to had institutional delivery compared to those women who did not faced complications. This finding is in agreement with other studies conducted in Ayssaita District, North East Ethiopia (28), and Benchi Maji, Southwest Ethiopia (27). This imply that those mothers who recognized the problem during pregnancy could have greater fear of the possible adverse birth outcomes. Therefore, they would be encouraged and motivated to deliver at health facility.

This study revealed that ANC visits was strong predictor of institutional delivery. Mothers with at least one ANC visit during the recent pregnancy were 16 times (AOR = 16.1, 95% CI:9.6–22) more likely to give birth at health facility compared to those who had no ANC follow up. This finding agreed with different studies conducted in Nepal, Tanzania and Ethiopia (29–34). This might be because the women would be counseled about complication readiness and birth preparedness during their ANC follow up which will encourage the women to deliver at health facility.

The odds of institutional delivery were 11 times (AOR = 11.1, 95% CI: 2.7–45.4) higher among mothers who had good knowledge compared to those who had poor knowledge. This finding was congruent with other studies in Ethiopia and some developing countries (29, 30, 32, 35). This might be because women with good knowledge about institutional delivery would know the benefits of institutional delivery and can predict the possible consequences of childbirth; Hence, they are more likely to give birth at the health facilities.

Women's attitude toward childbirth at health facility was another important predictor which affects institutional delivery service utilization. Women with positive attitude were 9.6 time (AOR = 9.6, 95% CI: 2.5–35.9) more likely to give birth at health facility compared to those with negative attitudes. The finding is similar with other studies conducted in Afar, Northeast Ethiopia, in Bench Maji zone, South West Ethiopia, and evidence from meta-analysis in Ethiopia (19, 27, 36). This can be justified as attitude is the most important aspect which affects the health care seeking habits of the women.

Finally, as limitation of this study recall bias may be introduced because the women were asked information about their exposure and delivery status retrospectively. Furthermore, since the study was cross sectional, it was not possible to establish the temporal relationship of variables.

## Conclusion

The magnitude of institutional delivery in the study area was low. Positive attitude toward institutional delivery, attending ANC, having good knowledge about institutional delivery, and facing a problem during pregnancy were strong predictors of facility-based childbirth. Community mobilization and health education on maternal health service is needed to increase uptake of antenatal care, ensure women's knowledge toward institutional delivery and promote benefits of institutional delivery to increase the number of institutional deliveries.

## Data availability statement

The original contributions presented in the study are included in the article/supplementary material, further inquiries can be directed to the corresponding author.

## Ethics statement

The studies involving human participants were reviewed and approved by Institutional Health Research Review Committee of Haramaya University, College of health and medical science. The patients/participants provided their written informed consent to participate in this study.

## Author contributions

TA is the primary author who initiated the study. IM and MD involved in the study design, supervision, and analysis. TM and AA drafted the manuscript. All authors have conceived, critically revised, read, and approved the final manuscript.
